# Can acoustic radiation force impulse imaging (ARFI) accurately diagnose renal masses? A protocol of systematic review and meta-analysis

**DOI:** 10.1097/MD.0000000000021500

**Published:** 2020-07-31

**Authors:** Jiang-Feng Wu, Li-Jing Ge, Xiao-Bo Ye, Yue Sun, Yun-Lai Wang, Zheng-Ping Wang

**Affiliations:** Department of Ultrasound, Dongyang People's Hospital, No. 60, Wuning West Road, Dongyang, Zhejiang, China.

**Keywords:** acoustic radiation force impulse imaging, diagnostic accuracy, meta-analysis, renal masses, systematic review

## Abstract

**Background:**

Renal masses are increasingly being discovered because of the wide accessibility of modern high resolution imaging procedures. Previous clinical studies have reported that acoustic radiation force impulse imaging (ARFI) is used for diagnosis of renal masses. However, no study has investigated this topic systematically. Therefore, this study will evaluate the diagnostic value of ARFI for the diagnosis of renal masses.

**Methods:**

A systematic search using the databases of Cochrane Library, EMBASE, Pubmed, WANGFANG, and China National Knowledge Infrastructure will be performed to identify studies in which patients with renal masses are assessed by ARFI. Two investigators will independently screen the literature and extract the data. Any discrepancies will be resolved via discussion with the senior author. Study quality will be assessed by the Quality Assessment of Diagnostic Accuracy Studies 2 tool, and pooled sensitivity and specificity of various ARFI findings for the diagnosis of renal masses will be determined. Summary receiver operating characteristic curve will be used to assess the overall performance of ARFI.

**Results:**

This study will evaluate the diagnostic value of ARFI for the diagnosis of renal masses through sensitivity, specificity, positive and negative likelihood ratio, and diagnostic odds ratio.

**Conclusion:**

This study will summarize the most recent evidence that focusing on the diagnosis of ARFI for renal masses.

**Study registration:**

INPLASY202060105.

## 1 Introduction

Renal masses are increasingly being discovered because of the wide accessibility of modern high resolution imaging procedures.^[1–3]^ Conventional ultrasound, computed tomography, and magnetic resonance imaging have been widely used to evaluate renal masses.^[4–6]^ However, conventional ultrasound often cannot produce the high level of information regarding renal masses that can be gained from computed tomography or magnetic resonance imaging.^[7–9]^

Acoustic radiation force impulse imaging (ARFI) as a new technology is able to differentiate between malignant and benign renal masses by providing shear wave velocity values to quantify the elasticity of renal masses.^[10–12]^ However, there are still various findings, and no systematic review has specifically assessed this issue.^[13–19]^ Therefore, we will carry out a systematic review and meta-analysis to synthesize the diagnostic value of ARFI for renal masses.

## 2 Methods

### 2.1 Objective

This study aims to evaluate the diagnostic value of ARFI in the diagnosis of renal masses.

### 2.2 Study registration

We have registered this study on INPLASY202060105. This meta-analysis will be conducted according to the Preferred Reporting Items for Systematic Reviews and Meta-Analyses (PRISMA) guidelines, which include 27 items and provide specific guidance for reporting of systematic reviews.^[19]^

### 2.3 Eligible criteria for including studies

#### 2.3.1 Type of studies

Randomized control trials and case control or prospective studies will be included.

#### 2.3.2 Type of participants

Studies involving patients with renal masses will be included.

#### 2.3.3 Type of index test

Index test: Studies using ARFI for the diagnosis of renal masses will be included.

Reference test: Studies using reference standards such as histopathology, cytopathology, and/or clinical follow-up will be included.

#### 2.3.4 Type of outcome measurements

The primary outcomes are sensitivity and specificity. The secondary outcomes are positive likelihood ratio, negative likelihood ratio, and diagnostic odds ratio.

### 2.4 Information sources and search strategy

#### 2.4.1 Electronic searches

Cochrane Library, EMBASE, Pubmed, WANGFANG, and China National Knowledge Infrastructure will be systematically searched to identify potentially eligible studies from inception to June 2020. Computer searches will be carried out using the Medical Subject Heading and keywords. Search strategy for Pubmed is presented in Table 1. Similar search strategies will be adapted to other electronic databases. There will be no limitations of language and publication status.

**Table 1:**
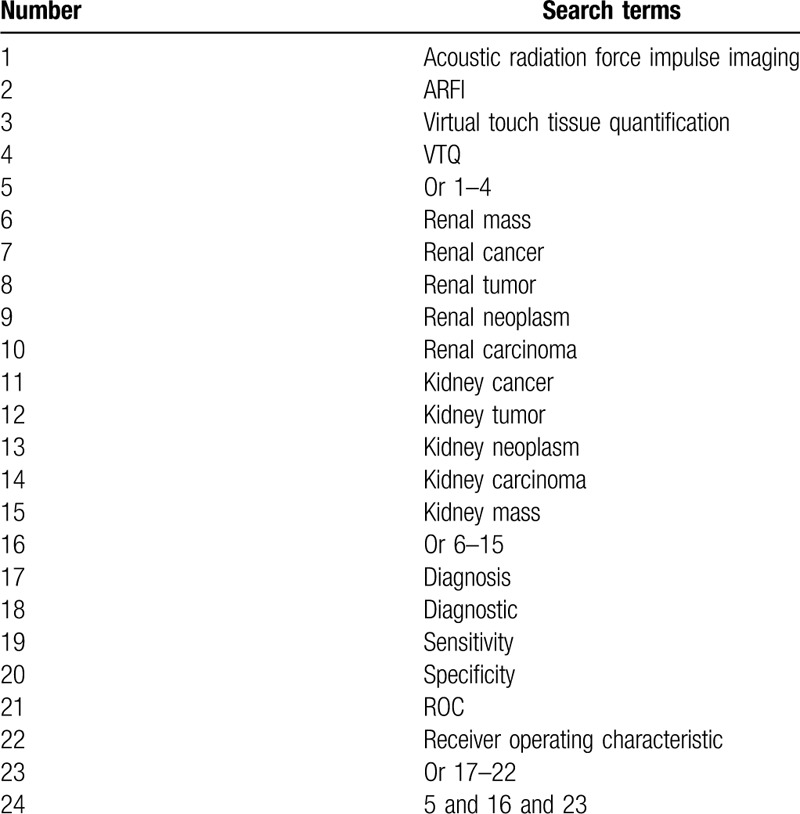
Search strategy applied in PubMed.

#### 2.4.2 Other resources

The bibliographies of identified studies and review articles will be manually screened to expand the number of eligible studies.

### 2.5 Data records and analysis

#### 2.5.1 Selection process of studies

We will export all articles from the searched results to the Endnote 7.0, and any duplicated studies will be removed. Two investigators will independently screen all literature to check whether they meet the specific inclusion criteria, and all irrelevant studies will be excluded. Then, full-text articles that meet the specific inclusion criteria will be obtained and judged. The whole process of study selection will be shown in a flowchart. Any divergences between the 2 investigators will be solved via discussion with a senior author when necessary. A list of excluded reasons alongside the rationale of their exclusion will be noted in an additional file.

#### 2.5.2 Data collection and management

Two researchers will independently extract the relevant data from the included studies using a predesigned data collection form. Any discrepancies will be resolved via discussion with the senior author. For eligible studies, the following items will be extracted: last name of the first author, year of publication, country, study type, blinding method, US equipment, sample size, mean age, gender, US diagnostic criteria, standard reference, tumor size, time between ARFI and the standard reference, true positives, true negatives, as well as false positives and false negatives of ARFI in the diagnosis of renal masses. If insufficient information occurs during the period of data collection, we will contact corresponding authors to obtain it.

### 2.6 Study quality assessment

The Quality Assessment of Diagnostic Accuracy Studies-2 tool will be utilized to evaluate the risk of bias and methodological quality by 2 investigators independently.^[20]^ Any discrepancies will be resolved via discussion with a senior author. The quality of each included study will be evaluated by an appraisal of the risk of bias of 4 domains and clinical applicability of three domains of the study characteristics. Four domains consisted of patient selection, index test, reference standard and flow and timing. Each domain will be evaluated for risk of bias, and the first 3 domains will be evaluated for applicability. The processing of the quality assessment will be performed utilizing RevMan 5.3 software (Nordic Cochrane Centre, Copenhagen, Denmark).

### 2.7 Statistical analysis

The present meta-analysis will be conducted by Stata 12.0 (Stata Corporation, College Station, TX). All statistical analyses will be performed by 1 investigator, who has experience in performing meta-analysis. The summary estimates of sensitivity, specificity, positive likelihood ratio, negative likelihood ratio, and diagnostic odds ratio with corresponding 95% confidence intervals will be calculated using a bivariate random effect model in the present analysis, which indicate the accuracy of ARFI in the diagnosis of renal masses. Meanwhile, the summary receiver operator curve will be constructed and the area under the curve (AUC) will be calculated. An AUC close to 0.5 shows a poor test, while an AUC of 1.0 demonstrates an excellent diagnostic test.^[21]^ We will be applying the spearman correlation analysis to determine whether a threshold effect is present, with *P* < .05 representing a threshold effect. The Cochrane *Q* test and the inconsistency index (*I*^2^) will be used to assess the heterogeneity among different studies with a *P*-value <0.1 or *I*^2^ > 50% considered significant for heterogeneity.^[22]^ Meta-regression analyses utilizing several covariates will be carried out to investigate the potential causes of heterogeneity.

### 2.8 Additional analysis

#### 2.8.1 Subgroup analysis

We will perform a subgroup analysis based on the characteristics of different studies or patients, comparators, and outcomes.

#### 2.8.2 Sensitivity analysis

We will plan to conduct a sensitivity analysis by removing low quality studies to check the robustness of outcome results.

#### 2.8.3 Reporting bias

We will check reporting bias using funnel plots and associated regression tests if necessary.^[23]^

### 2.9 Ethics and dissemination

This study does not need ethical approval because it will not analyze individual patient data. The results of this study will be submitted on a peer-reviewed journal.

## 3 Discussion

We will systematically and comprehensively search more electronic databases and other literature sources to avoid missing potential studies. Two independent investigators will conduct study selection, data extraction and study quality assessment. Any discrepancies will be resolved via discussion with the senior author. The study quality will be evaluated by using Quality Assessment of Diagnostic Accuracy Studies-2 tool.

To our knowledge, no studies have comprehensively evaluated the literature on renal masses diagnosis by using ARFI. Hence, we will carry out a systematic review and meta-analysis to synthesize the diagnostic accuracy of ARFI for renal masses.

## Author contributions

**Conceptualization:** Jiang-Feng Wu, Yun-Lai Wang, Li-Jing Ge, Xiao-Bo Ye, Zheng-Ping Wang.

**Data curation:** Jiang-Feng Wu, Yun-Lai Wang.

**Formal analysis:** Jiang-Feng Wu, Yun-Lai Wang, Zheng-Ping Wang.

**Funding acquisition:** Zheng-Ping Wang.

**Investigation:** Jiang-Feng Wu.

**Methodology:** Jiang-Feng Wu, Li-Jing Ge, Xiao-Bo Ye, Zheng-Ping Wang.

**Project administration:** Zheng-Ping Wang.

**Resources:** Zheng-Ping Wang.

**Software:** Jiang-Feng Wu, Yun-Lai Wang.

**Supervision:** Zheng-Ping Wang, Li-Jing Ge, Xiao-Bo Ye.

**Validation:** Jiang-Feng Wu, Yun-Lai Wang, Zheng-Ping Wang.

**Visualization:** Li-Jing Ge, Xiao-Bo Ye, Zheng-Ping Wang.

**Writing – original draft:** Jiang-Feng Wu, Li-Jing Ge, Xiao-Bo Ye, Zheng-Ping Wang.

**Writing – review & editing:** Jiang-Feng Wu, Yun-Lai Wang, Zheng-Ping Wang.

## References

[1] Kay FU Pedrosa I Imaging of solid renal masses. Urol Clin North Am 2018;45:311–30. 30031457 10.1016/j.ucl.2018.03.013 PMC6057157

[2] Bertolotto M Bucci S Valentino M Contrast-enhanced ultrasound for characterizing renal masses. Eur J Radiol 2018;105:41–8. 30017297 10.1016/j.ejrad.2018.05.015

[3] Garstka N Shariat SF Remzi M The evolving role of percutaneous biopsy in renal masses. Curr Opin Urol 2018;28:364–8. 29847524 10.1097/MOU.0000000000000513

[4] Woo S Suh CH Cho JY Diagnostic performance of CT for diagnosis of fat-poor angiomyolipoma in patients with renal masses: a systematic review and meta-analysis. AJR Am J Roentgenol 2017;209:W297–307. 28834444 10.2214/AJR.17.18184

[5] Ramamurthy NK Moosavi B McInnes MD Multiparametric MRI of solid renal masses: pearls and pitfalls. Clin Radiol 2015;70:304–16. 25472466 10.1016/j.crad.2014.10.006

[6] Krishna S Murray CA McInnes MD CT imaging of solid renal masses: pitfalls and solutions. Clin Radiol 2017;72:708–21. 28592361 10.1016/j.crad.2017.05.003

[7] Wang ZJ Westphalen AC Zagoria RJ CT and MRI of small renal masses. Br J Radiol 2018;91:20180131. 29668296 10.1259/bjr.20180131 PMC6221773

[8] van Oostenbrugge TJ Runneboom W Bekers E MRI as a tool to assess surgical margins and pseudocapsule features directly following partial nephrectomy for small renal masses. Eur Radiol 2019;29:509–16. 30043161 10.1007/s00330-018-5630-9 PMC6302880

[9] Zhou L Tang L Yang T Comparison of contrast-enhanced ultrasound with MRI in the diagnosis of complex cystic renal masses: a meta-analysis. Acta Radiol 2018;59:1254–63. 29363321 10.1177/0284185118755575

[10] Sun D Lu Q Wei C Differential diagnosis of <3 cm renal tumors by ultrasonography: a rapid, quantitative, elastography self-corrected contrast-enhanced ultrasound imaging mode beyond screening [published online ahead of print, 2020 Jun 9]. Br J Radiol 2020;Epub ahead of print. 10.1259/bjr.20190974 PMC7446022 32479108

[11] Thaiss WM Bedke J Kruck S Can contrast-enhanced ultrasound and acoustic radiation force impulse imaging characterize CT-indeterminate renal masses? A prospective evaluation with histological confirmation. World J Urol 2019;37:1339–46. 30324296 10.1007/s00345-018-2520-3

[12] Lu Q Wen JX Huang BJ Virtual touch quantification using acoustic radiation force impulse (ARFI) technology for the evaluation of focal solid renal lesions: preliminary findings. Clin Radiol 2015;70:1376–81. 26375726 10.1016/j.crad.2015.08.002

[13] Zaffanello M Piacentini G Bruno C Renal elasticity quantification by acoustic radiation force impulse applied to the evaluation of kidney diseases: a review. J Investig Med 2015;63:605–12. 10.1097/JIM.0000000000000186 25738649

[14] Guo LH Liu BJ Xu HX Acoustic radiation force impulse elastography in differentiating renal solid masses: a preliminary experience. Int J Clin Exp Pathol 2014;7:7469–76. 25550782 PMC4270564

[15] Göya C Daggulli M Hamidi C The role of quantitative measurement by acoustic radiation force impulse imaging in differentiating benign renal lesions from malignant renal tumours. Radiol Med 2015;120:296–303. 25096889 10.1007/s11547-014-0443-7

[16] Clevert DA Stock K Klein B Evaluation of Acoustic Radiation Force Impulse (ARFI) imaging and contrast-enhanced ultrasound in renal tumors of unknown etiology in comparison to histological findings. Clin Hemorheol Microcirc 2009;43:95–107. 19713604 10.3233/CH-2009-1224

[17] Fu NH Yang B Wei SP Acoustic radiation force impulse imaging in differential diagnosis of renal tumors. Chin J Med Imaging Technol 2013;29:621–4.

[18] Wen JX Xue LY Yan CJ Value of virtual touch tissue quantification in the diagnosis of renal tumors. Chin J Ultrasonogr 2013;22:38–41.

[19] Shamseer L Moher D Clarke M Preferred reporting items for systematic review and meta-analysis protocols (PRISMA-P) 2015: elaboration and explanation. BMJ 2015;350:g7647. 25555855 10.1136/bmj.g7647

[20] Whiting PF Rutjes AW Westwood ME QUADAS-2: a revised tool for the quality assessment of diagnostic accuracy studies. Ann Intern Med 2011;155:529–36. 22007046 10.7326/0003-4819-155-8-201110180-00009

[21] Hanley JA McNeil BJ The meaning and use of the area under a receiver operating characteristic (ROC) curve. Radiology 1982;143:29–36. 7063747 10.1148/radiology.143.1.7063747

[22] Higgins JP Thompson SG Deeks JJ Measuring inconsistency in meta-analyses. BMJ 2003;327:557–60. 12958120 10.1136/bmj.327.7414.557 PMC192859

[23] Deeks JJ Macaskill P Irwig L The performance of tests of publication bias and other sample size effects in systematic reviews of diagnostic test accuracy was assessed. J Clin Epidemiol 2005;58:882–93. 16085191 10.1016/j.jclinepi.2005.01.016

